# *Acacia senegal* Extract Rejuvenates the Activity of Phenicols on Selected *Enterobacteriaceae* Multi Drug Resistant Strains

**DOI:** 10.3390/antibiotics9060323

**Published:** 2020-06-13

**Authors:** René Dofini Magnini, Adama Hilou, Helana Millogo-Koné, Jean-Marie Pagès, Anne Davin-Regli

**Affiliations:** 1 Faculté de Pharmacie, UMR_MD1, U-1261, Aix-Marseille University, INSERM, SSA, IRBA, MCT, 13385 Marseille, France; dofinirene@gmail.com (R.D.M.); jean-marie.pages@univ-amu.fr (J.-M.P.); 2 Institut de Recherche en Sciences de la Santé (IRSS/CNRST), Département de Médecine et Pharmacopée Traditionnelle/ Pharmacie (MEPHATRA-PH), 03 BP 7047 Ouaga 03, Burkina Faso; hassmillogo@gmail.com; 3 Laboratoire de Biochimie et de Chimie Appliquée (LABIOCA), Université Ouaga I Pr Joseph Ki-Zerbo, 03 BP 848 Ouagadougou 03, Burkina Faso; hiloudio@gmail.com

**Keywords:** *Acacia senegal*, multiresistance, *Enterobacteriaceae*, efflux pumps, phenicols, antibiotic adjuvant

## Abstract

This study reported the phytochemical composition of two hydroethanolic extracts of *Acacia senegal* and *Acacia seyal* trees from Burkina Faso and their activities, alone or in combination with selected antibiotics, against multidrug resistant bacteria. High performance thin layer chromatography (HPTLC) method was used for phytochemical screening. Total phenolic and total flavonoid ant tannins in leaves extracts contents were assessed by spectrophotometric method. The minimal inhibitory concentrations (MICs) of plant extracts and antibiotics were determined using the microdilution method and *p*-iodonitrotetrazolium chloride. Combinations of extracts and antibiotics were studied using checkerboard assays. Screening revealed the presence of phenolic compounds, flavonoids, and tannins in the hydroethanolic extract (HE) of the leaves. The HE of *A. seyal* showed the highest total phenolic (571.30 ± 6.97 mg GAE/g), total flavonoids (140.41 ± 4.01 mg RTE/g), and tannins (24.72 ± 0.14%, condensed; 35.77 ± 0.19%, hydrolysable tannins). However, the HE of *A. senegal* showed the lowest total phenolic (69.84 ± 3.54 mg GAE/g), total flavonoids (27.32 ± 0.57 mg RTE/g), and tannins (14.60 ± 0.01%, condensed; 3.09 ± 0.02%, hydrolysable). The MICs for HE and antibiotics were in the range of 2–512 and 0.008–1024 mg/L, respectively. All tested HE presented an MIC greater than 512 mg/L except HE of *A. senegal*. The lowest MIC value (128 mg/L) was obtained with HE of *A. senegal* against *Klebsiella aerogenes* EA298 and *Escherichia coli* AG100A. Interesting restoring effects on chloramphenicol and florphenicol activity were detected with alcoholic extracts of *A. senegal* against resistant *E. coli* and *K. aerogenes* strains that overproduce AcrAB or FloR pumps. The adjuvant effect of HE of *A. senegal* suggests that the crude extract of leaves could be a potential source of molecules for improving the susceptibility of bacteria to phenicols antibiotics.

## 1. Introduction

Traditional medicine occupies an important place in the care of African populations; it represents an easy and inexpensive therapeutic means in regions where access to health infrastructures (hospitals, dispensaries, pharmacies) are underdeveloped and/or expensive for the patient family. Consequently, about 80% of the population in developing countries uses traditional medicinal plants for primary care management [1,2]. Today, documentation of the medicinal uses of plants is imperative, following the rapid disappearance of some of these plants species due to human activities [3]. The overuse and the misuse of antibiotics has led to the emergence of multiresistant bacteria, inducing worrying public health problem. Consequently, the implementation of appropriate strategies to bypass therapeutic impasses are urgently needed, and medicinal plants are attractive ways for the research and development of original alternate therapeutic molecules [4]. Medicinal plants extracts usually contain mixtures of different chemical compounds that can act individually, additively, or synergistically to combat multi-drug resistant (MDR) infections [5]. Between 1981 and 2019, in the area of cancer and antibacterial therapies, more than a thousand new drug molecules were characterized, from which 60% were issued from natural products [6]. Moreover, many available antimicrobial drugs used in therapeutics are derived from bacterial or fungal molecules [7]. Medicinal plants from the African continent have been previously studied for biological properties, and they exhibit interesting antibacterial activity [8]. Preclinical data on traditional uses show that *A. senegal* treats respiratory tract infections, diarrhea, stomach aches, hemorrhoids, ulcers, trypanosomiasis, sexually transmitted diseases, wounds, malaria, abscesses, and boils [9,10,11,12]. Another traditional healer treats dysentery, gastrointestinal pain, leprosy, nervous sensory and digestive disorders, toothache, rheumatism, stomach ulcers, jaundice, intestinal parasites, and syphilis by roots, leaves, barks, and gum of *A. seyal* [10,13,14]. In recent studies, extracts of *A. senegal* and *A. seyal* have shown good activity against many agents responsible for infectious diseases [12,15,16,17] and have interesting chemical components such as phenolic compounds, flavonoids, tannins, or terpenes recognized as having a strong antibacterial potential [8,18]. There is no study regarding possible adjuvant propriety of their extracts and their capability to permeabilize the bacterial membrane. In this study, we aim to evaluate the anti-bacterial potential of two Burkinabè medicinal plants, namely *Acacia Senegal* and *Acacia seyal*, against Gram-negative *Enterobacteriaceae* strains. We investigated the activity spectrum of these extracts for restoring the efficacy of phenicols antibiotic families in MDR bacteria.

## 2. Materials and Methods

### 2.1. Plants Material and Extraction

The collection of plant samples was done in early June and August in the area of Saaba in Gonsé, mapped about twenty kilometers from Ouagadougou (Burkina Faso). The plants were certified by Pr A. Ouedraogo of Botany Section, University Joseph KI-ZERBO of Ouagadougou, and voucher specimens were deposited under references numbers 6896/17257 (*A. senegal*) and 6897/17258 (*A. seyal*). The different parts of each plant were dried under ventilation deprived of sunlight for 21 days in a drying room. The samples were then ground to fine powder using a blade mill (Gladiator Est., 1931 Type BN 1 Mach 40461 1083). The vegetable powders were placed in freezer bags and stored at 4 °C in the freezer for further use. 

#### 2.1.1. Hydroethanolic Extracts (HE)

Extracts were prepared by taking 100 g of *A. senegal* and *A. seyal* leaf and root bark powder and soaking it in ether petroleum (500 mL) for 24 h in the first step. The residue was filtered by Whatman filter N°1, and the marcs were dried and soaked again in ethanol (1000 mL) at 70% (V/V) overnight. Rotary vacuum evaporator at 50 °C removed the extraction solvent. This hydroethanolic extracts were freeze dried and store at 4 °C until use [19,20]. 

#### 2.1.2. Aqueous Extract

One hundred grams of powder of *A. senegal* and *A. seyal* root bark were boiled in 1000 mL of distilled water for 30 min. After cooling, the extracts were first filtered on a nylon cloth and then centrifuged at 2000 rpm for 5 min. Supernatants were collected and then lyophilized and weighed. The extracts were stored at 4 °C until use [21,22].

#### 2.1.3. Soxhlet Extraction

From each plant, 50 g of dried powdered leaves were put into the extraction filter that was covered with cotton and then transferred into a Soxhlet apparatus with a series of four solvents of increasing polarity. Extraction solvents (500 mL) were added to each flask, which was connected to the extractor. Each extraction was performed in triplicate during 8 h. The temperature of extraction corresponded with the boiling point of the different solvent in use. Each solvent was concentrated at 40 °C using rotary evaporator, and their excess was eliminated by drying at 40 °C for 1 h. The crude extracts were weighed before being stored at 4 °C for further analysis [23,24]. 

### 2.2. Phytochemical Composition

#### 2.2.1. High-Performance Thin Layer Chromatography (HPTLC) Screening

Phytochemical screening of samples extracts was performed on 20 cm × 10 cm silica gel 60 F HPTLC (glass) plate (Merck, Darmstadt, Germany). Two µL of each extract were applied as 5 mm bands with a semi automatic plate spotter (CAMAG, Linomat V, Switzerland) set to dispense along a line 10 mm from the bottom edge of the plate. The distance between tracks was 10 mm. Distances from left and right edge of the plate were 20 mm. The plates were placed in a 20 × 20 cm vee bottomed TLC tank (saturation time 30 min) containing ethyl acetate:formic acid:acetic acid:water (100:11:11:26) and ethyl acetate:water:methanol:n hexane (11.9:1.6:1.4:3.5), respectively, for flavonoids and tannins. The developed plates were then dried with an air dryer (cold air) for 5 min. Concerning flavonoids, the plate was heated at 105 °C for 2 min and sprayed with the Neu reagent. Evaluation was performed under UV 366 nm. As for the tannins, the plate was sprayed with a 2% FeCl_3_ reagent. Evaluation was performed under white light [25].

#### 2.2.2. Determination of Total Phenolic Content

Different plant extracts (25 µL, 100 µg/mL in methanol) were mixed with Folin Ciocalteu reagent (105 µL, 0.2 N) and 5 min later with sodium bicarbonate (100 μL, 75 g/L). After 1 h incubation, the absorbance of each mixture was measured (spectrophotometer UV, Shimadzu) at 760 nm against a blank. A standard calibration curve was plotted using Gallic acid (Y = 0.0664X − 0.0009; R^2^ = 0.9991). Polyphenol content was expressed as mg of Gallic acid equivalent per g of extract (mg GAE/g).

#### 2.2.3. Determination of Total Flavonoids Content

The extract was prepared at a concentration of 1 mg/mL in methanol. Then, 1 mL of this extract was mixed with 3 mL of double-distilled water followed by 0.3 mL of NaNO_2_ at 5% (m/v); 5 min later, 0.3 mL of AlCl_3_ 10% (m/v) was added. The whole was incubated at room temperature for 6 min. Subsequently, 1 mL of NaOH 1 N was added. The absorbance of the mixture was measured at 510 nm using a UV spectrophotometer (Shimadzu). Calculation was based on a calibration curve obtained with increasing concentration of rutin solution following the same procedure. The flavonoid content of the sample, expressed as milligrams of rutin equivalent per g of plant material (mg RT/g), was obtained by relating the absorbance read on the calibration curve [26].

#### 2.2.4. Determination of Tannin Content

##### Hydrolyzable Tannins

One mL of the extract and 3.5 mL of the reagent (FeCl_3_ 10^−2^ M in HCl 10^−3^ M) were mixed. The absorbance of the mixture was measured at 660 nm after 15 s [27]. The hydrolysable tannins content T (%) was determined using the following formula: T (%) = A × PM × V × FD/ε mole × P

A = absorbance, Ɛ mole = 2169 (for gallic acid), PM = weight of gallic acid (170.12 g/mol), V = volume of extract, P = sample weight and FD = dilution factor.

##### Condensed Tannins

The reagent was vanillin 1% (1 g of vanillin dissolved in 100 mL of 70% sulfuric acid); 2 mL of this reagent was added to 1 mL of extract. The absorbance of the mixture was measured at 500 nm after incubation of 15 min in a water bath at 20 °C [28]. The condensed tannins content T (%) was determined using the following formula: T (%) = (5.2 × 10^-2^ × A × V)/P

5.2 × 10^−2^ = equivalent constant of cyanidine, A = absorbance, V = extract volume and P = sample weight.

### 2.3. Bacterial Strains 

Phenotypic and/or genotypic characteristics for identification and changes in membrane permeability of strains used in this study are presented in Table 1. Bacteria were routinely grown at 37 °C on Luria-Bertani agar (LB).

### 2.4. Determination of Minimal Inhibitory Concentration (MIC) of the Plant Extracts

Direct antibacterial activity was determined by the microdilution method as described [36]. In practice, 20 mg of each plant extract were dissolved in 100 μL of DMSO and then completed to 5 mL with broth to obtain a stock concentration of 4 mg/mL (2% DMSO). Then, this solution was diluted to obtain an entry concentration of 512 mg/L with a concentration of 0.2% in DMSO. This solution was transferred to the wells of the first column of the 96-well plates (200 μL/well) for serial dilution with Muller-Hinton II (MHII). One hundred (100 μL) of the bacterial suspension (5 × 10^5^ CFU/mL) was added to each well to obtain a final volume of 200 μL, with a final DMSO concentration of 0.1%. The plates were incubated for 18 hours at 37 °C without agitation with closed lid. The minimal inhibitory concentrations (MICs) of samples were observed after the addition 40 µL of 0.2 mg/mL of iodonitrotetrazolium chloride. MIC values were recorded as the lowest concentration of the sample that totally inhibited bacterial growth. The reference antibiotics used in the present work included chloramphenicol, florfenicol, thiamphenicol, ciprofloxacin, cefepime, and ceftazidime (Sigma-Aldrich). Objectives were to detect molecules that were able to modulate the bacterial permeability of the bacterium in terms of penetration and efflux.

### 2.5. Combination with Antibiotics 

The activity of the various combinations, antibiotics, and extracts of *A. senegal* and *A. seyal* were determined by the checkerboard test [37]. Serial dilutions of two antimicrobial agents were mixed so that each row (and column) contained a fixed concentration of the first agent (extract) and an increasing concentration of the second (antibiotics). The concentrations of the extracts were distributed vertically from 1 to 512 mg/L, and the antibiotic was dispatched from 1 to 1024 mg/L horizontally according to the sensibility of each bacteria. Thus, 190 µL of fresh bacterial suspension prepared in MH2 broth (5 × 10^5^ CFU/mL) were added to each well. The first column was used to determine the MIC of the extract alone. The microplates were closed and incubated for 18 h at 37 °C. The MICs of samples were observed after the addition 40 µL of 0.2 mg/mL *p*-iodonitrotetrazoluim chloride (INT). MIC values were recorded as the lowest concentration of the sample that totally inhibited bacterial growth.

### 2.6. Outer Membrane Permeation Assay

An overnight culture of *K. aerogenes* 289 was diluted 100-fold into 10 mL MH2 broth. This strain was used since it normally expresses β-lactamase that is used to monitor outer membrane integrity [38]. After reaching an optical density (OD) of 600 nm of 0.5, cells were recovered by centrifugation (4000× *g* for 20 min) and washed twice in 20 mM potassium phosphate buffer (PPB pH 7) supplemented with 1 mM MgCl_2_ to reach an OD of 0.375. Then, 50 μL of each concentration was added to 100 μL of the cell suspension, yielding final concentrations ranging from 256 to 8 μg/mL. Then, 50 μL of nitrocefin was added to obtain a final concentration of 50 μg/mL. Absorbance at 490 nm was monitored by spectrophotometry using an Infinite M200 microplate reader (Tecan) over 60 min at 37 °C. Tazobactam (10 mg/mL) and clavulanic acid (1 mg/mL) were used as inhibitors and PPB as negative controls. Experiments were performed in triplicate. The effect on membrane permeability for extract was determined using the slope in the linear range and compared to the slope obtained with polymyxin B (used at 200 μM according to [37]).

### 2.7. Statistical Analysis

Quantification results for secondary compounds are expressed as mean ± SEM (n = 3). The analysis was performed using Graph Pad Prism 5 (Graph Pad Software, San Diego, CA, USA) followed by Dunett’s multiple comparison tests. Statistical procedures were performed using a p value less than 0.05.

## 3. Results 

### 3.1. Phytochemical Screening by HPTLC

**Figure 1:**
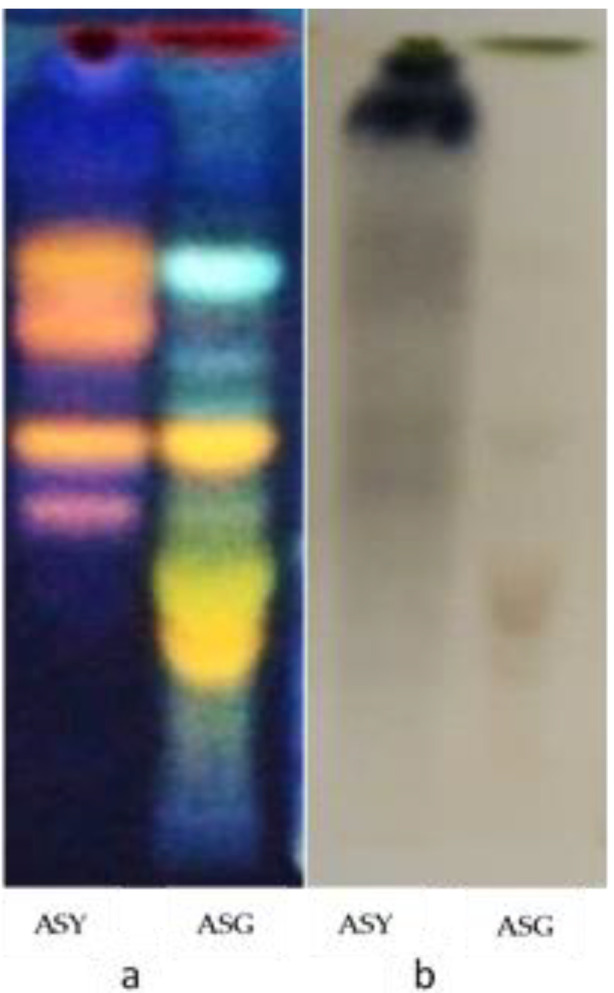
(**a**) Presence of flavonoids sprayed by Neu’s reagent at UV 365 nm (orange, yellow and green spots); (**b**) tannins sprayed with FeCl3 2% (blue-black, brown spots); ASY: hydroethanolic leaves of *Acacia seyal*; ASG: hydroethanolic leaves of *Acacia senegal.*

Phytochemical screening of (HE) of *A. senegal* and *A. seyal* resulted in the chromatoplates (Figure 1). The HPLC chromatographic profile showed blue, green, orange, yellow, and fluorescence spots under ultraviolet 366 nm, characterizing the presence of flavonoids in the HE of two extracts (Figure 1a). In addition, after spraying with the 2% FeCl_2_ reagent, the chromatoplate presented a much more intense brown spot in the HE of *A. seyal* compared to the HE of *A. senegal* in the visible section (Figure 1b). The brown spots were characteristic of the tannins. 

### 3.2. Phytochemical Contents 

**Figure 2:**
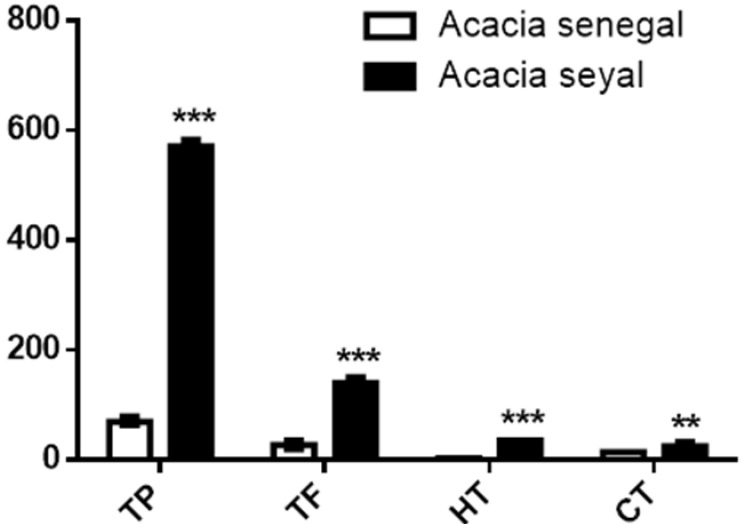
Phytochemical content of *A. senegal* and *A. seyal*. TP: Total phenols (exprimed in mg GAE/g; TF: Total flavonoids (exprimed in mg RTE/g); HT: Hydrolysable tannins; CT: Condensed tannins (exprimed in %). ** indicates statistically significant difference in values (*p* < 0.01); *** indicates statistically significant difference in values (*p* < 0.001).

Determination of phenolic, flavonoids, and tannins contents of HE of *A. senegal* and *A. seyal* leaves was performed by spectrophotometric method. Results of phenolic compounds, flavonoids, and tannins contents are presented in Figure 2. The total polyphenol and flavonoid contents of the hydroethanolic extract of *A. seyal* was 571.30 ± 6.97 mg GAE/g and 140.41 ± 4.01 mg RTE/g, respectively, and showed a very significant difference (*p* < 0.001) from the extract of *A. senegal* with respective values of 69.84 ± 3.54 mg GAE/g and 27.32 ± 0.57 mg RTE/g. In addition, the HE from the leaves of *A. seyal* showed higher percentages of condensed and hydrolyzable tannins (24.72 ± 0.14 and 35.77 ± 0.19, respectively) compared to the HE of *A. senegal* with percentages of 14.60 ± 0.01 and 3.09 ± 0.02, respectively. A statistical difference of *p* < 0.001 for condensed tannins and *p* < 0.01 for hydrolyzable tannins was obtained. 

### 3.3. Antibacterial Effect of the Various Extract 

Table 2 presents the MICs for the antibiotics tested in this study. The susceptibility data concerning the extracts showed that almost all of the extracts tested had a weak antibacterial activity (Table 3). The best activity was recorded with hydroethanolic (HE) extract from the leaves of *A. senegal*, with MIC values ranging from 128 to 512 mg/L. The lowest MIC value (128 mg/L) was recorded with HE from *A. senegal* leaves against *K. aerogenes* EA298 and *E. coli* AG100A, followed by *E. coli* AG100 and *K. aerogenes* ATCC 11296 with an MIC of 256 mg/L. The antibacterial activity of a plant extract is considered significant when the MIC values are less than 100 mg/L, moderate when the MIC comprises between 100 < MIC < 625 mg/L, and low when the MIC > 625 mg/L [39]. Interestingly, the activity (MIC of 128 mg/L and 256 mg/L) was observed with HE from *A. senegal* against *Ea*298, *Ea* ATCC 11296, AG100, and AG100A. 

### 3.4. Combination with Different Antibiotics

A preliminary study performed against different strains cited in Table 1 allowed us to select two out of fourteen extracts at the appropriate subinhibitory concentrations for further studies. All the extracts were combined separately to six antibiotics (CIP, CHL, FLOR, THIAM, CEF, and CAZ) to evaluate their possible synergetic effects. Table 4 indicated that HE from the leaves of *A. senegal* and *A. seyal* were able to improve the antibacterial activity of the phenicols group against *K. aerogenes, K. pneumoniae* MDR strains, and *E. coli* strains. With 128 mg/L of HE leaf extract combined with the chloramphenicol, a significant decrease in the MIC with Ea289 (MIC CHL = 1024 mg/L), Ea CM64 (MIC CHL = 512 mg/L), AG100 (MIC CHL = 8 mg/L), and AG102 (MIC CHL = 64 mg/L) was observed and reached 128, 32, 2, and 4 mg/L, respectively. Significant effects on MIC were also observed when HE from the leaves of *A. senegal* was combined with florfenicol and thiamphenicol (Table 4). For *A. seyal*, HE at a concentration of 512 mg/L increased the susceptibility to chloramphenicol of AG100 (MIC = 8), AG102 (MIC = 64 µ/mL), *Ea* 289 (MIC = 1024 mg/L), and *Ea* CM64 (MIC = 512 mg/L) to 2, 16, 256, and 128 mg/L, respectively. Interestingly, susceptibility to other antibiotics (CAZ, CIP, and CEF) assayed in this study was not modified at all.

### 3.5. Effect on Membrane Permeability

The permeability test of the outer membrane was carried out with the most effective HE from the leaves of *A. senegal*. The nitrocefin hydrolysis was measured in the absence or the presence of an increased amount of extract to detect a direct effect on the membrane permeability. Polymyxin B that permeabilizes the bacterial outer membrane (OM) of Gram-negative bacteria was used as internal control and used as standard to compare the activity of various plants extracts [37,40]. The HE had a weak effect on the OM permeability at low concentrations (8–32 mg/L) compared to polymyxin B. A more significant effect was observed at 64 mg/L (12% of polymyxin effect) and increased at 128 mg/L (Table 5). At 256 mg/L, the OM permeability was seriously altered by HE *A. senegal* and may explain some antibacterial effects observed for high concentrations.

### 3.6. Combination Tests with Phenicols

The data in Table 4 identified the effects of plants extract on the susceptibility towards selected antibiotics. The HE from the leaves of *A. senegal* and *A. seyal* had interesting adjuvant effects by combining with chloramphenicol and florfenicol, which belong to the phenicol family. The MICs of the combinations indicated that HE from *A. senegal* has a better adjuvant effect on some strains (AG100, AG102, *Ea*289, and *Ea* CM64) than methanolic extract (Table 6). The presence of *A. senegal* HE (16 mg/L) in combination with chloramphenicol increased the susceptibility, e.g., MICs decreased from 64 to 4 mg/L for the AG102 and the *Ea* CM64 strains. These values are quite similar to those obtained with florfenicol. With methanolic extract, it was necessary to use 64 mg/L to observe the same effects. The direct MICs values obtained with methanolic extract from the leaves of *A. seyal* were greater than 512 mg/L (Table 3). The combination of the extract (256 mg/L) with chloramphenicol and florfenicol showed a decrease of the MIC from 512 to 128 mg/L for chloramphenicol and from 256 to 64 mg/L for florfenicol with strain CM64. The results were similar with the AG102 and the AG100 strains (data not shown). The associations of methanolic extracts of *A. senegal* and *A. seyal* (at 128 and 256 mg/mL, respectively) with other selected antibiotics (flerofloxacin, erythromycin, norfloxacin, and ciprofloxacin) showed no effect (Table S1).

### 3.7. FloR Pump and A. senegal HE

In order to define the effect of HE on phenicols transport, we measured the variation of susceptibility conferred by the FloR pump in the AG100A strain [41]. It was interesting to note that HE was able to potentialize the activity of chloramphenicol and florfenicol on the strain overproducing FloR (Table 7) In contrast, no significant modification was detected on the strain carrying only the empty plasmid. Moreover, the MICs to ceftazidime and norfloxacin were not modified at a concentration increasing phenicols susceptibility (Table 7). This suggests that HE was able to mask/protect the phenicols against the expel activity due to FloR pump.

## 4. Discussion

Phytochemical screening indicated the presence of several classes of secondary metabolites such as polyphenols, flavonoids, and tannins in the HE of *A. senegal* and *A. seyal* leaves. In vitro experiments showed that molecules belonging to these classes can be active on pathogenic microorganisms [8]. The results showed that the HE of *A. senegal* leaves increased the susceptibility of strains producing efflux pumps (Ea289, EaCM64, AG102, and AG100) for two antibiotics (chloramphenicol and florfenicol) with gains between 2 and 64 fold compared to assays performed without extracts. The adjuvant capability of natural extracts or compounds against resistant bacteria were already reported [41,42]. Previous studies have shown that HE from the root wood of *A. senegal* had a moderate direct activity against selected bacteria [43]. The same authors reported that dichloromethane extract of *A. senegal* root heartwood exhibited antibacterial activity against *E. coli* and *S. aureus*. The hexane fraction of the trunk bark of *A. senegal* is active against respiratory pathogenic bacteria such as *K. pneumonia* and *Streptococcus pneumoniae*. This observation would be due to the presence of tannins, steroids, cardiac glycosides, flavonoids, saponins, and alkaloids in *A. senegal* [44]. Plant extracts action can induce the alteration of the bacterial membrane facilitating the penetration of antibiotics into the Gram-negative bacteria that increases the intracellular concentration of antibiotics closed to the target [42,45,46]. As described, Epicatechin 3-gallate and caffeic acid disrupt the outer membrane of Gram-negative bacteria and increase the permeability of *Pseudomonas aeruginosa* [47]. A recent study showed that *A. nilotica* extracts disrupt the cell wall and the cytoplasmic membrane of *Salmonella* and *E. coli,* causing the leakage of cytoplasmic components [48]. Another hypothesis is that some plant-derived compounds may alter the efflux of usual antibiotics [43,49]. HE of *A. senegal* can permeabilize the OM when used at high amounts, but—interestingly—at low concentrations, it is able to potentialize mainly the phenicol antibacterial activity, suggesting an original synergy with this antibiotic group. Many extracts or compounds from plants have been recognized as efflux pump inhibitors when used as adjuvants in combination with specific antibiotics [43,50]. Flavonoids, polyphenols, terpenoids, and alkaloids potentially interact with antibiotics to improve their action on the target [51]. For example, conessin, a steroidal alkaloid, has been described as a MexAB-OprM efflux pump inhibitor in *P. aeruginosa*, restoring the antibiotic activity [52]. A chalcone (polyphenol) extracted from *Dalea versicolor* (Fabaceae) potentiates the action of berberine, erythromycin, and tetracycline on strains of *S. aureus* overexpressing NorA [53]. A Kaempferol derivative from *Persea lingue* showed inhibitory activity on the NorA efflux pump of *S. aureus* and restored ciprofloxacin susceptibility [54]. Carnosol and carnosic acid (diterpenoids) from *Rosmarinus officinalis* have also shown a modulating effect on erythromycin and tetracycline resistance in mutant strains of *S. aureus* [55]. In the present study, *A. senegal* extracts impair the activity of FloR pump without changing the susceptibility of other antibiotics, and this may suggest an affinity for this kind of transporter. This suggests that the extract contains compounds that may disturb the membrane stability and/or a compound targeting a selective efflux activity, thus increasing the antibiotic activity [56]. Importantly, the extract used at the same concentrations in combination showed no effect on other antibiotic groups. This indicates that one or more molecule present in the HE extract may have an affinity for the recognition/binding site of phenicol in the efflux process. This activity is also observed when FloR is overexpressed in an AcrAB minus strain; the extract is able to reverse the resistance level afforded by this selective pump against phenicols without altering other susceptibility. Recent phytochemical work on the leaves of *A. senegal* indicated the presence of several chemical groups such as sterols and steroids, phenols, alkaloids, flavonoids, leucoanthocyanins, anthocyanins, volatile oils, amino acid proteins, and carbohydrates [16]. Our preliminary study revealed the presence of flavonoids, phenolic, and tannins in the leaves of *Acacia senegal* and corroborates with previous plant studies. The presence of such phytochemical compounds in the extract of *A. senegal* can explain the antibacterial activities reported here. Phenicols are broad-spectrum drugs exhibiting a relative toxicity that seriously compromises its use in human medicine. For instance, in meningitis caused by *Haemophilus influenzae* or *Neisseria meningitidis* (in cases of penicillin allergy, for example), it diffuses easily into blood and tissues [57,58]. Combination with HE from the leaves of *A. senegal* could be an alternative to both restoring bacterial susceptibility to phenicols and to reducing the dose required for treatment, thus decreasing a hazard for adverse side effects.

## 5. Conclusions

This study explored the efficacy of the HE of *A. senegal* alone and in combination with antibiotics. Synergistic effects indicated that antibacterial combinations were more effective on characterized resistant strains. Many teams are looking for molecules that can inhibit the efflux pump. The tested combination of natural products with chloramphenicol and florfenicol pave the way for the development of efficient agents active against MDR bacteria. The discovery of natural compounds that impair the membrane-associated mechanisms of resistance in Gram-negative bacteria will increase the chances to combat resistant pathogens and stimulate the activity of usual antibiotics at low concentrations.

## Figures and Tables

**Table 1:** Bacterial strains used in this study.

Strains	Description	Reference
***E. coli***		
AG100	Parental *E. coli* K-12 Porin +; basal efflux	[29]
AG100A	AG100 acrAB::Kan^r^ non (AcrAB-); Porin +	[29]
AG102	AG100 overexpressing the AcrAB pump, Porin +	[29]
AG1004 plasmid AG100A FloR	wid plasmid without *floR* Expressing *floR*	[30] [30]
***K. aerogenes***		
Ea CM64	CHL variant obtained from ATCC 13048 overexpressing the AcrAB pump; Porin+	[31]
Ea 289	KAN-sensitive derivative of EA27, Porin-	[32]
Ea 298	EA289 tolC::Kan^r^; Porin-	[32]
EaATCC 15038	Porin +; Normal efflux	[33]
***K. pneumoniae***		
ATCC 12296	Reference strain; Porin +; Normal efflux	[34]
KP 45	Porin +; Normal efflux	[35]
KP55	Clinical MDR isolate; Tet^r^ Amp^r^ Atm^r^ Cef^r^; Porin-; Normal efflux	[35]

**Table 2:** Minimal inhibitory concentrations (MICs, mg/L) of the different antibiotics against the tested bacterial species MIC.

Antibiotics	*E. coli*				*K. aerogenes*			*K. pneumoniae*		
	AG100	AG100A	AG102	Ea 289	Ea298	Ea ATCC 15038	CM64	KP45	KP55	KPATCC 11296
Chloramphenicol	8	1	64	1024	32	4	512	8	16	8
Florfenicol	16	1	128	256	4	8	512	8	8	16
Thiamphenicol	256	16	1024	>1024	>1024	64	>1024	128	256	256
Ciprofloxacin	0.03	0.008	0.25	32	4	<0.125	0.5	0.06	4	0.06
Cefepime	<0.125	<0.125	0.25	2	8	<0.125	0.25	<0.125	16	<0.125
Ceftazidime	0.5	0.25	2	>64	>64	0.03	2	0.25	>64	0.5

**Table 3:** Minimal inhibitory concentrations (MICs, mg/L) of the different extracts against the tested bacterial species.

Plant Extract	*E. coli*			*K. aerogenes*				*K. Pneumoniae*		
	AG100	AG100A	AG102	Ea 289	Ea298	Ea ATCC 15038	CM64	KP45	KP55	KP ATCC 11296
**Soxhlet extraction** ***Acacia senegal*** **(leaves)**										
Hexane extract	>512	>512	>512	>512	>512	>512	>512	>512	>512	>512
Dichloromethane extract	>512	>512	>512	>512	>512	>512	>512	>512	>512	>512
Ethyl acetate extract	>512	>512	>512	>512	>512	>512	>512	>512	>512	>512
Methanolic extract	>512	>512	>512	>512	>512	>512	>512	>512	>512	>512
***Acacia seyal*** **(leaves)**										
Hexane extract	>512	>512	>512	>512	>512	>512	>512	>512	>512	>512
Dichloromethane extract	>512	>512	>512	>512	>512	>512	>512	>512	>512	>512
Ethyl acetate extract	>512	>512	>512	>512	>512	>512	>512	>512	>512	>512
Methanolic extract	>512	>512	>512	>512	>512	>512	>512	>512	>512	>512
**Cold-Maceration**										
***Acacia senegal***										
Hydroethanolic extract (**leaves**)	**256**	**128**	**>512**	**>512**	**128**	**256**	>512	>512	**512**	>512
Hydroethanolic (**Root Bark**)	>512	>512	>512	>512	>512	>512	>512	>512	>512	>512
***Acacia seyal***										
hydroethanolic (**Leaves**)	>512	>512	>512	>512	>512	>512	>512	>512	>512	>512
Hydroethanolic (**Root Bark**)	>512	>512	>512	>512	>512	>512	>512	>512	>512	>512
**Aqueous**										
***Acacia senegal***										
Root bark	>512	>512	>512	>512	>512	>512	>512	>512	>512	>512
***Acacia seyal***										
Root bark	>512	>512	>512	>512	>512	>512	>512	>512	>512	>512

ASGMOH: Methanolic extract of Acacia senegal leaves; ASYMOH: Methanolic extract of Acacia seyal leaves, ASGHE: Hydroethanolic extract of Acacia senegal leaves.

**Table 4:** MIC of antibiotics associated to extracts at various concentrations.

				*E. coli*			*K. pneumoniae*			*K. aerogenes*			
Plants	Extracts	ATBs	Extracts (mg/L)	AG100	AG100A	AG102	KP45	KP55	KP ATCC 11296	Ea289	Ea298	Ea ATCC 15038	Ea CM64
*A. senegal*	Decoction root bark	CHL	0	8	1	64	8	16	8	1024	32	4	512
			512	8	1	64	8	16	8	1024	64	4	512
			128	16	1	64	8	16	8	1024	32	4	512
			16	16	1	64	8	16	8	1024	32	4	512
		CIP	0	0.03	0.008	0.25	0.06	4	0.06	64	4	<0.125	0.5
			512	0.03	0.008	0.25	0.03	4	0.06	64	8	<0.125	1
			128	0.03	0.008	0.25	0.06	4	0.06	64	4	<0.125	1
			16	0.03	0.008	nd	nd	4	Nd	64	4	<0.125	0.5
		CAZ	0	0.5	0.25	2	0.25	>64	0.25	>64	>64	0.03	2
			512	1	0.5	2	0.5	>64	0.25	>64	>64	0.0.3	2
			128	0.5	0.25	2	0.5	>64	0.25	>64	>64	0.03	2
			16	1	<0.125	nd	nd	>64	nd	>64	>64	>2	2
	Hydroethanolic root bark	CHL	0	8	1	64	8	16	8	1024	32	4	512
			512	8	1	64	8	16	8	1024	32	4	512
			128	8	1	64	8	16	8	1024	32	4	512
			16	8	1	64	8	16	16	1024	32	4	512
		CIP	0	0.03	0.008	0.25	0.06	4	0.06	64	4	<0.125	0.5
			512	0.03	0.008	0.25	0.125	4	0.06	64	4	<0.125	0.5
			128	0.03	0.008	0.25	0.06	4	0.125	64	4	<0.125	0.5
			16	0.03	0.008	0.25	0.06	4	0.06	64	4	<0.125	0.5
		CEF	0	0.06	<0.03	0.5	<0.125	16	<0.125	2	8	<0.125	0.25
			512	0.06	<0.03	0.5	0.5	8	<0.125	4	8	<0.125	0.25
			128	0.06	<0.03	0.5	0.06	8	<0.125	2	16	<0.125	0.25
			16	0.06	<0.03	0.5	0.06	8	<0.125	2	16	<0.125	0.25
	hydroethanolic leaves extract	CHL	0	8	1	64	8	16	8	1024	32	4	512
			512	**1**	**<0.125**	**2**	**2**	**1**	**2**	**64**	**4**	**<2**	**16**
			128	**2**	**1**	**4**	**2**	**4**	**2**	**128**	**16**	**<2**	**32**
			16	8	2	64	8	16	4	1024	32	4	512
		CIP	0	0.03	0.008	0.25	0.06	4	0.06	64	4	<0.125	0.5
			512	0.03	<0.008	0.125	0.06	1	0.06	64	1	<0.125	<0.125
			128	0.03	<0.008	0.25	0.06	2	0.06	64	2	<0.125	<0.125
			16	0.03	0.008	0.25	0.06	4	0.03	64	2	<0.125	0.5
		CAZ	0	0.5	0.25	2	0.25	>64	0.25	>64	>64	0.03	2
			512	0.25	**<0.125**	0.5	0.5	>64	2	>64	>64	0.03	1
			128	0.5	0.25	1	0.5	>64	1	>64	>64	0.03	2
			16	0.25	0.25	1	0.25	>64	0.5	>64	>64	0.03	2
		FLOR	0	16	1	128	8	8	16	256	4	8	512
			512	**0.5**	**<0.125**	**4**	**2**	**2**	**2**	**8**	**0.25**	**2**	**32**
			128	**2**	**0.5**	**8**	**4**	**2**	**2**	**16**	**1**	**2**	**32**
			16	16	1	128	8	8	8	256	2	8	256
		THIAM	0	256	16	1024	128	256	256	>1024	>1024	64	>1024
			512	**8**	**<0.125**	**128**	**128**	**64**	**64**	>1024	**4**	64	512
			128	128	4	256	128	64	128	>1024	1024	128	1024
			16	128	16	1024	128	128	256	>1024	>1024	>1024	>1024
A. *seyal*	Decoction root bark	CHL	0	8	1	64	8	16	8	1024	32	4	512
			512	8	1	32	8	16	2	1024	32	4	256
			128	8	1	64	8	16	8	1024	32	4	512
			16	8	1	64	16	>64	16	1024	32	4	1024
		CIP	0	0.06	0.008	0.25	0.06	4	0.06	64	4	<0.125	0.5
			512	0.06	0.008	0.25	0.06	4	0.06	64	8	<0.125	0.5
			128	0.06	0.008	0.5	0.06	4	0.06	64	4	<0.125	0.5
			16	0.06	0.008	0.25	0.5	8	0.06	64	4	<0.125	0.5
		CAZ	0	0.5	0.25	2	0.25	>64	0.25	>64	>64	0.03	2
			512	0.5	0.5	2	2	>64	0.125	>64	>64	0.03	2
			128	0.5	0.5	2	1	>64	0.5	>64	>64	0.03	2
			16	1	0.25	2	1	>64	0.5	>64	>64	0.06	4
	Hydroethanolic root bark	CHL	0	8	1	64	8	16	8	1024	32	4	512
			128	8	<0.125	64	8	16	8	1024	32	4	512
			16	16	<0.125	64	8	16	8	1024	64	8	512
			1	8	0.25	64	8	16	8	1024	32	**512**	512
		CIP	0	0.03	0.008	0.25	0.06	4	0.06	64	4	<0.125	0.5
			128	0.03	0.008	0.25	0.06	4	0.06	64	4	<0.125	0.5
			16	0.03	0.008	0.25	0.06	4	0.06	64	2	<0.125	0.5
			1	0.03	0.008	0.5	0.06	4	0.06	64	2	<0.125	0.5
		CEF	0	<0.125	<0.125	0.25	2	8	0.125	2	16	<0.125	0.25
			128	<0.125	<0.125	0.5	0.125	8	0.125	4	8	<0.125	0.25
			16	<0.125	<0.125	0.25	0.125	8	0.125	4	8	<0.125	0.25
			1	0.06	<0.125	0.5	0.25	16	0.125	4	16	<0.125	0.25
	Hydroethanolic leaves extract	CHL	0	8	1	64	8	16	8	1024	32	4	512
			512	**2**	**1**	**16**	**4**	**4**	**4**	**256**	**64**	**2**	**128**
			128	4	1	32	4	16	8	1024	128	4	512
			16	8	1	64	4	16	8	1024	128	4	512
		CIP	0	0.03	0.008	0.25	0.06	4	0.06	64	4	<0.125	0.5
			512	0.03	0.016	0.25	0.06	8	0.06	>64	4	<0.125	0.5
			128	0.03	0.008	0.125	0.06	4	0.125	64	8	<0.125	0.5
			16	0.03	0.008	0.125	0.06	4	0.125	64	4	<0.125	0.5
		CAZ	0	0.5	0.25	2	0.25	>64	0.25	>64	>64	0.03	2
			512	0.5	0.25	0.25	0.25	>64	0.5	>64	>64	0.03	1
			128	0.5	0.25	1	0.25	>64	0.5	>64	>64	0.03	2
			16	0.5	0.25	0.5	0.25	>64	0.25	>64	>64	0.06	1

**FLOR**: florfenicol; **CHL**: chloramphenicol; **CAZ**: ceftazidime; **CEF**: cefepime; **THIAM**: thiamphenicol; **CIP**: ciprofloxacin; **ATB**_S_: antibiotics.

**Table 5:** Effect of *A. senegal* hydroethanolic extract on bacterial outer membrane of *K. aerogenes* 289 expressed as percentage of polymyxin B activity.

	Permeating Effect					
**Concentration extract (mg/L)**	**256**	**128**	**64**	**32**	16	8
% of polymyxin B	50 (±18 *)	31 (±13 *)	12 (±4 *)	7.3 (±2 *)	2 (±1 *)	2 (±1 *)

* The interval was calculated from three independent tests performed in triplicates.

**Table 6:** Adjuvant activity of leaves extract of *A. senegal,* MIC in mg/L (Gain). Bacteria strains, MIC (mg/L) of antibiotics in the presence and absence of the extract.

*Hydroethanolic Extract of Acacia Senegal (mg/L)*								*Methanolic Extract Acacia Senegal (mg/L)*				
ATB	Strain	MIC ATB	MIC ASG	8	16	32	64	MIC ASG	8	16	32	64
**CHL**	***E. coli***											
	AG100	8	256	8 (-)	4 (2)	2 **(4)**	1 **(8)**	>256	16 (-)	8 (-)	4 (2)	2 **(4)**
	AG100A	1	128	1 (-)	2 (-)	1 (-)	0.5(2)	>256	1 (-)	1 (-)	1 (-)	1 (-)
	AG102	64	>256	64 (-)	16 **(4)**	4 **(16)**	4 **(16)**	>256	64 (-)	64 (-)	32 (2)	8 **(8)**
	***K. aerogenes***											
	Ea289	1024	>256	1024 (-)	512 (2)	64 **(16)**	64 (16)	>256	1024 (-)	1024 (-)	512 (2)	128 **(8)**
	Ea298	32	128	32 (-)	32 (-)	16 (2)	4 **(8)**	>256	32 (-)	32 (-)	32 (-)	16 (2)
	Ea ATCC 15038	4	256	4 (-)	2 (2)	1 **(4)**	1 **(4)**	>256	4 (-)	4 (-)	2 (2)	2 (2)
	Ea CM64	512	>256	512 (-)	128 **(4)**	16 **(32)**	16 **(32)**	>256	512 (-)	256 (2)	256 (2)	32 **(16)**
**FLOR**	***E. coli***											
	AG100	16	256	8 (2)	4 **(4)**	2 **(8)**	2 **(8)**	>256	32 (-)	16 (-)	8 (2)	4 **(4)**
	AG100A	1	128	1 (-)	**1 (-)**	1 (-)	0.25 **(4)**	>256	1 (-)	1 (-)	1 (-)	1 (-)
	AG102	64	>256	128 (-)	**32 (2)**	8 **(8)**	4 **(16)**	>256	64 (-)	64 (-)	32 (2)	16 **(4)**
	***K. aerogenes***											
	Ea289	128	>256	32 (4)	64 (2)	16 **(8)**	8 **(16)**	>256	128 (-)	64 (2)	64 (2)	32 **(4)**
	Ea298	4	128	4 (-)	2 (2)	2 (2)	1 **(4)**	>256	4 (-)	4 (-)	4 (-)	2 (2)
	Ea ATCC 15038	4	256	4 (-)	2 (2)	2 (2)	2 (2)	>256	4(-)	4 (-)	4 (-)	2 (2)
	Ea CM64	256	>256	256 (-)	128 (2)	16 **(16)**	16 **(16)**	>256	256 (-)	256 (-)	64 **(4)**	32 **(8)**

MIC: Minimal inhibitory concentration, ATB: Antibiotic, ASG: A. senegal, CHL: chloramphenicol, FLOR: florfenicol.

**Table 7:** FloR expression and phenicols susceptibility in absence or in presence of HE extract.

ATBs	Strain	MIC (mg/L)		*Conc. HE Acacia senegal (mg/L)*					
	***E. coli***	**ATBs**	**ASG**	**10**	**20**	**30**	**40**	**50**	**64**
**CHL**	AG100 A	2	128	2	2	2	2	2	2
	Plasmid	1	128	1	1	1	1	1	1
	FloR	8	128	8	4	4	2	2	1
**FLOR**	AG100 A	1	128	1	1	1	1	1	1
	Plasmid	1	128	1	1	1	1	1	1
	FloR	4 - 2	128	4	4	1	1	1	0.5
**THIAM**	AG100 A	16	128	16	8	8	8	4	4
	Plasmid	16	128	16	8	8	8	4	4
	FloR	512	128	256	256	128	64	64	32
**CAZ**	AG100 A	0.25	128	0.25	0.25	0.25	0.25	0.25	0.25
	Plasmid	0.25	128	0.25	0.25	0.25	0.25	0.25	0.25
	FloR	0.25	128	0.25	0.25	0.25	0.25	0.25	0.125
**NOR**	AG100 A	0.03	128	0.03	0.03	0.03	0.03	0.03	0.03
	Plasmid	0.016	128	0.016	0.016	0.016	0.016	0.016	0.016
	FloR	0.016	128	0.016	0.016	0.016	0.016	0.016	0.016

**MIC**: Minimal inhibitory concentration, **ATB**: antibiotic, **ASG**: *A. senegal, **CHL***: chloramphenicol, FLOR: florfenicol, **THIAM**: thiamphenicol, **CAZ**: ceftazidime, **NOR**: norfloxacin.

## Supplementary Materials

The following are available online at https://www.mdpi.com/2079-6382/9/6/323/s1, **Table S1**: Antibiotic resistance modulatory activity of methanolic extract of *Acacia senegal* leaves (μg /mL).

## Abbreviations

DMSO: dimethyl sulfoxyde; HE: hydroethanolic extract; LB: Luria Bertani: MDR: multi drug resistant; MH: Mueller Hinton; MIC: minimal inhibitory concentration; OM: outer membrane. FLOR: florfenicol; CHL: chloramphenicol; CAZ: ceftazidime; CEF: cefepime; THIAM: thiamphenicol; CIP: ciprofloxacin; ATBs: antibiotics.
