# Supplementary material for: Acacia senegal Extract Rejuvenates the Activity of Phenicols on Selected Enterobacteriaceae Multi Drug Resistant Strains
Source: Antibiotics (Basel). 2020 Jun 13;9(6):323. doi: 10.3390/antibiotics9060323 (PMC7344600; doi:10.3390/antibiotics9060323)
Supplement: Supplementary file 1 [file antibiotics-09-00323-s001.pdf]

Table S1. Antibiotic resistance modulatory activity of methanolic extract of *Acacia senegal* leaves (µg /mL).

ATBs	Strains	MIC ATBs	MIC ASG	8	16	32	64
	E. coli						
NOR	AG100	0,25	>256	0.25 (-)	0.25 (-)	0.25 (-)	0.25 (-)
	AG100A	0,03	>256	0.03 (-)	0.03 (-)	0.03 (-)	0.03 (-)
	AG102	1	>256	1 (-)	1 (-)	1 (-)	1 (-)
FLERO	AG100	0,125	>256	0.125 (-)	0.125 (-)	0.125 (-)	0.06 (2)
	AG100A	0,06	>256	0.06 (-)	0.06 (-)	0.06 (-)	0.06 (-)
	AG102	0,5	>256	0.5 (-)	0.5 (-)	0.5 (-)	0.25 (2)
CIP	AG100	0,03	>256	0.03 (-)	0.03 (-)	0.06 (-)	0.03 (-)
	AG100A	0,008	>256	0.008 (-)	0.008 (-)	0.008 (-)	0.008 (-)
	AG102	0,125	>256	0.125 (-)	0.125 (-)	0.125 (-)	0.125 (-)
	K. aerogenes						
ERY	Ea 289	256	>256	256 (-)	256 (-)	256 (-)	128 (2)
	Ea 298	4	>256	8 (-)	4 (-)	4 (-)	2 (2)
	Ea ATCC 15038	128	>256	128 (-)	128 (-)	128 (-)	32 **(4)**
